# Supplementary material for: A validation of Illumina EPIC array system with bisulfite-based amplicon sequencing
Source: PeerJ. 2021 Feb 10;9:e10762. doi: 10.7717/peerj.10762 (PMC7881719; doi:10.7717/peerj.10762)
Supplement: Supplemental Information 1 — The Christchurch Health and Developmental Study cohort selected for analysis by BSAS. Cases = cannabis and tobacco users; Controls = never cannabis users. [file peerj-09-10762-s001.pdf]

Supplementary Table 1- The Christchurch Health and Developmental Study cohort selected for analysis by BSAS. Cases = cannabis and tobacco users; Controls = never cannabis users.

	Cases	Controls
Individuals	n=44	n=38
Gender		
Male	84%	76%
Female	16%	24%
Ethnicity		
European	73%	89%
Other	27%	11%
Socioeconomic status		
Professional/managerial	20%	37%
Clerical/technical	41%	39.%
Semi-skilled/unskilled	39%	24%
Tobacco smoking status		
Never	9%	92%
Occasional	4%	3%
Regular	87%	5%
