# Supplementary material for: A validation of Illumina EPIC array system with bisulfite-based amplicon sequencing
Source: PeerJ. 2021 Feb 10;9:e10762. doi: 10.7717/peerj.10762 (PMC7881719; doi:10.7717/peerj.10762)
Supplement: Supplemental Information 2 — Forward and reverse primers used to target validation sites using bisulfite amplicon sequencing CpG sites including an Illumina overhang sequence. [file peerj-09-10762-s002.pdf]

Supplementary Table 2- Forward and reverse primers used to target validation sites using bisulfite amplicon sequencing CpG sites including an Illumina overhang sequence

Primer name	Illumina probe ID	Bisulfite converted primer (including Iluminia overhang sequence)
SLC17A7_F	Cg 02624701	TCGTCGGCAGCGTCAGATGTGTATAAGAGACAGGTATTAGAAGATTTYGAAGTTGTTT
SLC17A7_R	Cg 02624701	GTCTCGTGGGCTCGGAGATGTGTATAAGAGACAGAAAATAAACCTATTCTCTCC
AHRR_F	Cg 05575921	TCGTCGGCAGCGTCAGATGTGTATAAGAGACAGGTTTTTTTGGTGTGGTTTTA
AHRR_R	Cg 05575921	GTCTCGTGGGCTCGGAGATGTGTATAAGAGACAG ACCACCATCTTATCTTATTT
ITPR1_F	Cg 08987995	TCGTCGGCAGCGTCAGATGTGTATAAGAGACAG GATGGAATTTATTAGTGTTT
ITPR1_R	Cg 08987995	GTCTCGTGGGCTCGGAGATGTGTATAAGAGACAGTAAAACACAACCCATTATCT
MAGI2_F	Cg 21121803	TCGTCGGCAGCGTCAGATGTGTATAAGAGACAGAGTTTAATTGAGTGTTTTTGAGG
MAGI2_R	Cg 21121803	GTCTCGTGGGCTCGGAGATGTGTATAAGAGACAG ACCCATTTTATTTATACCTTT
EHMT2_F	Cg 07829740	TCGTCGGCAGCGTCAGATGTGTATAAGAGACAG AGAGGGGTTTAAATTTAAGTTTG
EHMT2_R	Cg 07829740	GTCTCGTGGGCTCGGAGATGTGTATAAGAGACAG CTAATAAATCACATATCTCC
PPM1L_F	Cg 26406186	TCGTCGGCAGCGTCAGATGTGTATAAGAGACAG AATGTTAGTTGAATAAGTGG
PPM1L_R	Cg 26406186	GTCTCGTGGGCTCGGAGATGTGTATAAGAGACAGCCACAAAAATACTCTAAAAAC
DPP10_F	Cg 05868547	TCGTCGGCAGCGTCAGATGTGTATAAGAGACAG TTAAGGGAAGAAAGAAATGT
DPP10_R	Cg 05868547	GTCTCGTGGGCTCGGAGATGTGTATAAGAGACAGCCTCTATAACAACATTTACTCAA
NIPAL4_F	Cg 17695979	TCGTCGGCAGCGTCAGATGTGTATAAGAGACAGGTTGGGAGAATTTATTTTTAGAG
NIPAL4_R	Cg 17695979	GTCTCGTGGGCTCGGAGATGTGTATAAGAGACAGATATACCTATCACCAACTTC
CHD7_F	Cg 19926587	TCGTCGGCAGCGTCAGATGTGTATAAGAGACAG TTTTAAAAGGATTTAAGGTAATG
CHD7_R	Cg 19926587	GTCTCGTGGGCTCGGAGATGTGTATAAGAGACAGACTAACACAAAACAACCCAAT
PRDM5_F	Cg 01118724	TCGTCGGCAGCGTCAGATGTGTATAAGAGACAGATTTAAAAATGGTTGTGGTGAAG
PRDM5_R	Cg 01118724	GTCTCGTGGGCTCGGAGATGTGTATAAGAGACAGTCCACTCATTACTCATATACTA
Cg11977356_F	Cg 11977356	TCGTCGGCAGCGTCAGATGTGTATAAGAGACAGGGAGGTGAGATGTTTTAATAATT
Cg11977356_R	Cg 11977356	GTCTCGTGGGCTCGGAGATGTGTATAAGAGACAGAATAAACTATAATCATACCCCTC
Cg09078959_F	Cg 09078959	TCGTCGGCAGCGTCAGATGTGTATAAGAGACAGGTTTGAAAAGGGGAAATTTA
Cg09078959_R	Cg 09078959	GTCTCGTGGGCTCGGAGATGTGTATAAGAGACAGACACTTAATAAAACACCAATC
Cg00571101_F	Cg 00571101	TCGTCGGCAGCGTCAGATGTGTATAAGAGACAGTGATAGGATATAAGAAGAAAGTA
Cg00571101_R	Cg 00571101	GTCTCGTGGGCTCGGAGATGTGTATAAGAGACAGTCTACTTCAACCTAAAACAA
Cg11293828_F	Cg 11293828	TCGTCGGCAGCGTCAGATGTGTATAAGAGACAGTAGGGGGTTAGAGTATTTATTTT
Cg11293828_R	Cg 11293828	GTCTCGTGGGCTCGGAGATGTGTATAAGAGACAGTTTTACTTTACTTAACTTCTCCC
Cg01614625_F	Cg 01614625	TCGTCGGCAGCGTCAGATGTGTATAAGAGACAGATGGAATTAGAAATTTTGGG
Cg01614625_R	Cg 0161462	GTCTCGTGGGCTCGGAGATGTGTATAAGAGACAG CCTCTCCATTTTATTTCTTTAA
