# Supplementary figures and images for: A validation of Illumina EPIC array system with bisulfite-based amplicon sequencing
Source: PeerJ. 2021 Feb 10;9:e10762. doi: 10.7717/peerj.10762 (PMC7881719; doi:10.7717/peerj.10762)

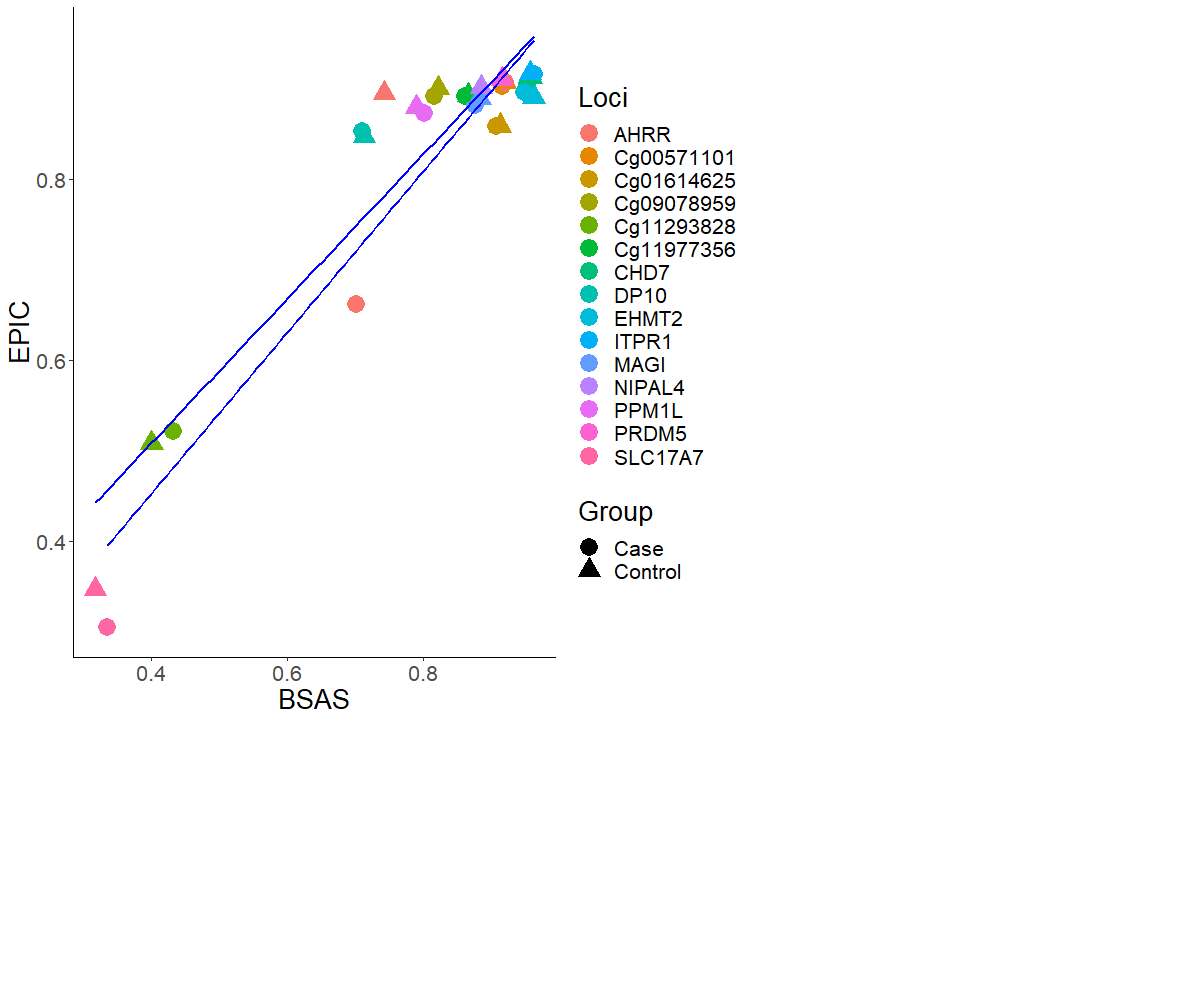

Supplement: Supplemental Information 3 — Scatter plot with a linear regression of the β values at each locus for BSAS and EPIC array plotted against each other. Colours represent the loci of interest, with the shapes representing the case and controls. There are two regression lines: A represents the correlation between cases with an adjusted R2 = 0.8878 and B represents controls with R2 = 0.8683. [file peerj-09-10762-s003.png]

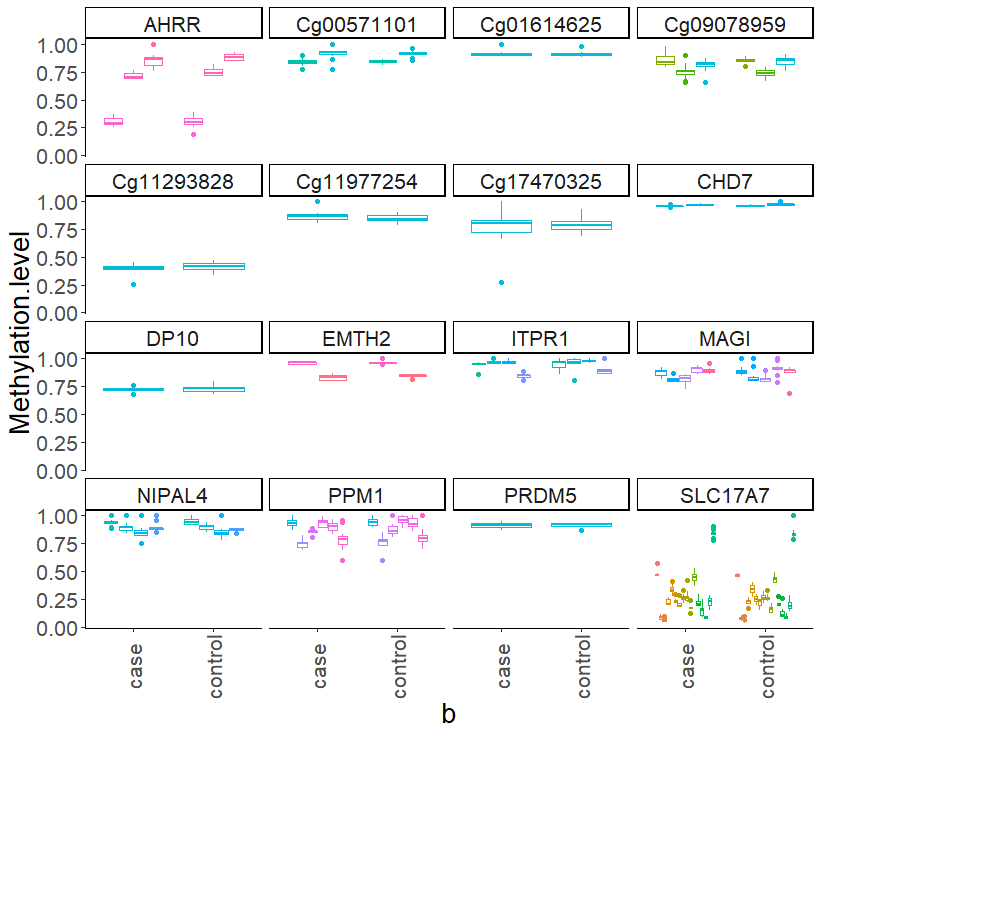

Supplement: Supplemental Information 4 — Average DNA methylation between cannabis cases compared to controls across all CpGs that were assessed. A differing number of CpG sites are found within each of the 16 gene regions assessed using BSAS. *Cg17470325 wasn’t included in the previous analysis, due to not passing QC using the noob normalisation method for EPIC array analysis. No methylation differences were observed using BSAS for this CpG site either. [file peerj-09-10762-s004.png]
